# The Atomic Structure and Mechanical Properties of ZIF-4 under High Pressure: Ab Initio Calculations

**DOI:** 10.3390/molecules28010022

**Published:** 2022-12-20

**Authors:** Zuhao Shi, Kaiyi Weng, Neng Li

**Affiliations:** 1 State Key Laboratory of Silicate Materials for Architectures, Wuhan University of Technology, Wuhan 430070, China; 2 Shenzhen Research Institute, Wuhan University of Technology, Shenzhen 518000, China; 3 State Center for International Cooperation on Designer Low-Carbon & Environmental Materials (CDLCEM), School of Materials Science and Engineering, Zhengzhou University, Zhengzhou 450001, China

**Keywords:** zeolitic imidazolate frameworks, elastic properties, high pressure, Ab initio calculation

## Abstract

The effects of pressure on the structural and electronic properties and the ionic configuration of ZIF-4 were investigated through the first-principles method based on the density functional theory. The elastic properties, including the isotropic bulk modulus *K*, shear modulus *G*, Young’s modulus *E,* and Poisson’s ratio *ν* of the orthorhombic-type structure ZIF-4 were determined using the Voigt–Reuss–Hill averaging scheme. The results show that the ZIF-4 phase is ductile according to the analysis of *K/G* and Cauchy pressure. The Debye temperatures obtained from the elastic stiffness constants increase with increasing pressure. Finally, the pressure-dependent behaviors of the density of states and ionic configuration are successfully calculated and discussed.

## 1. Introduction

Zeolitic imidazolate frameworks (ZIFs) are a unique class of porous hybrid materials known as a subset of metal–organic frameworks (MOFs), and have been studied extensively over the past decade due to their potential applications in gas sorption and separation, catalysis, drug delivery, and sensing applications [1,2,3,4,5]. Especially in the field of environmental protection, ZIFs and their composite structures have also shown considerable potential in research into the capture of greenhouse gases recently [6,7,8,9]. The ZIF structure consists of tetrahedral metal ions, typically Zn^2+^ or Co^2+^, linked by organic bridging ligands, which are derived from imidazolate anions (Im = C_3_N_2_H_3_^−^) [10]. They have significantly softer mechanical properties compared with their inorganic cousins and may structurally collapse upon heating [11,12,13,14], pressurization [15,16,17], ball-milling [18,19,20], or under an electric field [21] to form amorphous frameworks [22], which possess the same short-range connectivity as their crystalline counterparts [23,24]. Among the various ZIFs, ZIF-4 (Zn(C_3_N_2_H_3_^−^)_2_) has attracted lots of interest because of its unique behaviors, such as remarkable structural flexibility [25], and has great potential in the field of small-molecule adsorption and separation [26,27]. In particular, the complex phase transition behavior of ZIF-4 under high pressure [28] and its vitrification have attracted a great deal of attention from researchers [29]. Bennett et al. showed that amorphization occurs at various hydrostatic pressure values depending on the presence or absence of solvent molecules in the pores of the material, as well as the nature of the hydrostatic medium. The evacuated ZIF-4 demonstrated amorphization at very low pressure (0.35 GPa). At the same time, the presence of DMF (dimethylformamide, C_3_H_7_NO) molecules in its pores could shift amorphization to higher pressure and lead to an intermediate monoclinic crystalline phase (ZIF-4-I) [15]. Recent in situ XRD (X-ray diffraction) studies have shown that ZIF-4 undergoes multiple crystal–crystal transitions before undergoing an irreversible amorphous transition [28]. The elastic behavior of ZIF-4 and its high-pressure phase ZIF-zni is well explained by the simulation research of Tan et al. [30]. Furthermore, the op–cp phase transitions (i.e., breathing transitions) of ZIF-4 driven by mechanical pressure were identified and taken into account. The change in pore size suggested potential applications of the gas storage of this functional material [31]. Lately, Vervoorts et al. have applied a cutting-edge HPXRD (high-pressure X-Ray diffraction) setup to probe the response of ZIF-4 at low hydrostatic pressures. An initial correlation between the mechanical properties of ZIF-4 and mid-range structural parameters, such as free volume, is presented [32]. 

The structure–properties relationship of ZIF-4 is plagued by several obstacles, such as small pressure needs and small structure characterization. Computation simulation provides a powerful tool to probe the microscopic details of structure evaluation under pressure [33,34]. For complex structures such as ZIFs, ab initio methods without empirical parameters can reveal deeper correlations between electronic structural and mechanical properties [35,36,37,38]. To our knowledge, there is no systematic study of the mechanical properties–structure relationship of ZIF-4 with varying pressure up to now. 

**Figure 1:**
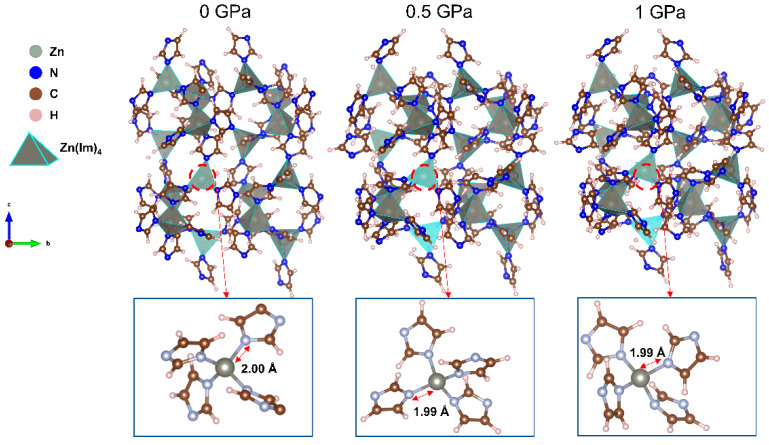
Structures of ZIF-4 with varying pressure.

Figure 1 shows the schematic structure of ZIF-4 at *p* = 0, 0.5, and 1 GPa. Additionally, the insert diagrams show the average Zn-N bond lengths at varied pressures. The variation in the local environment of Zn is very limited, as can be seen by the change in Zn-N bond length, and Zn(Im)_4_ still has a tetrahedral structure at different pressures. Changes in mechanical properties under pressure can often be explained by structural features in the mid-range. To further investigate the response to different pressures, the present work is conducted to clarify the phase stability, elastic constants, and electronic properties of ZIF-4 under high pressure of up to 1 GPa.

## 2. Results

**Figure 2:**
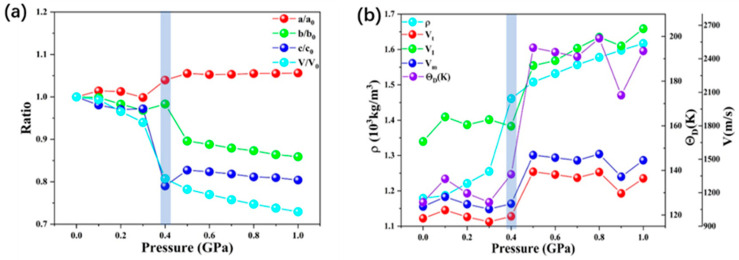
(**a**) Pressure dependence of lattice parameter ratios and volume ratio. (**b**) The pressure dependence of density (*ρ*), average sound velocity (*V_t_*), longitudinal sound velocity (*V_I_*), average wave velocity (*V_m_*), and Debye temperature (*θ_D_*) of ZIF-4.

The model of the ZIF-4 structure is firstly fully optimized under 0 GPa. The calculated lattice parameters are very close to the available experimental data [15] and other DFT calculation works [39]. From Table 1, the structure at zero pressure has a difference of about 3% on the b-axis. The main reason is the overestimation of the lattice parameters by the GGA-PBE method [40,41]. Such systematic errors would not have a practical impact on the reliability of this work, considering that all calculations were performed under the same parameters and criteria. The pressure dependences of lattice parameter ratios and volume ratios are illustrated in Figure 2a. The corresponding results are listed in Table 1. The a-axis slightly increases as the pressure increases from 0.3 to 0.5 GPa, indicating that ZIF-4 has negative linear compressibility (NLC) in this pressure range. A recent study has shown that ZIF-75 exhibits similar negative linear compressibility under 0.1 GPa [42]. The NLC effect has also been investigated in more MOF-related studies, which are thought to be caused by the contraction of metal–ligand bonds and the tilting of ligands [43]. Drastic volume change occurs in structures at 0.3 to 0.4 GPa, where the cell volume is reduced from 4224.366 Å^3^ to 3629.236 Å^3^. This mutation corresponds to a change in the structural characteristics of the middle range. It has also been shown experimentally that at such low pressures, the major cause of ZIF-4 compression is the contraction of free volume in the mid-range structure [32].

The calculated values of transverse sound velocity V_t_, longitudinal sound velocity *V_l_*, average wave velocity *V_m_*, and Debye temperature *θ_D_* from 0 GPa to 1 GPa are listed in Table S1 (see Supplementary Materials) and Figure 2b. The Debye temperature increases with increasing pressure. It is difficult to compare calculated and experimental data since there are no experimental data regarding ZIF-4 under the high pressure of the Debye temperature. The present results can be served as a prediction for future experiments. In Debye’s theory, the *θ_D_* is the temperature of a crystal’s highest normal mode of vibration, i.e., the highest temperature that can be achieved due to a single normal vibration. The longitudinal sound velocity *V_l_* is insensitive to pressure until 0.4 GPa, and thereafter it displays a positive pressure dependence. Similarly, the transverse sound velocity *V_t_* and average wave velocity *V_m_* also undergo a transition at 0.4–0.5 GPa. Combined with the dramatic decrease in structural parameters occurring at 0.4 GPa, we suggest that this may correspond to the reversible phase transition process of ZIF-4 found in the experiments [15]. Combined with the experimentally measured phenomenon of increasing sound velocity during the vitrification of ZIF-4, we suggest that this change correlates with the degree of ZIF-4 amorphization. The discussion of the structural transformation and mechanical property changes in this pressure range will be continued below.

The elastic stiffness constants of solids are essential parameters because they can provide a link between the mechanical properties and dynamic information concerning the forces operating in solids, especially for the stability and stiffness of materials. Elastic constants reflect the resistance of a crystal to externally applied stress. Study of the elastic properties of materials is essential to understand the chemical bonds and the cohesion of materials. Thus, it is significant to investigate elastic constants to understand the mechanical properties of the ZIF-4 phase at different pressures. The elastic constants of a single crystal were obtained through geometry optimization. The ZIF-4 phase belongs to the orthorhombic crystal, which has nine independent elastic constants, i.e., *C*_11_, *C*_22_, *C*_33_, *C*_44_, *C*_55_, *C*_66_, *C*_12_, *C*_13_, and *C*_23_. In this work, the calculated elastic constants of ZIF-4 at different pressures are listed in Table S2. According to Mouhat et al., the necessary and sufficient Born criteria of mechanical stability for orthorhombic structures are as follows [44]:

(1) $$C_{11}>0;\;C_{11}C_{22}>C_{12}{}^{2}\;$$

(2) $$C_{11}C_{22}C_{33}+2C_{12}C_{13}C_{23}-C_{11}C_{23}{}^{2}-C_{22}C_{13}{}^{2}-C_{33}C_{12}{}^{2}>0\;$$

(3) $$C_{44}>0;C_{55}>0;C_{66}>0\;$$

In our results, all structures are mechanically stable up to 1 GPa according to these stability criteria. 

**Figure 3:**
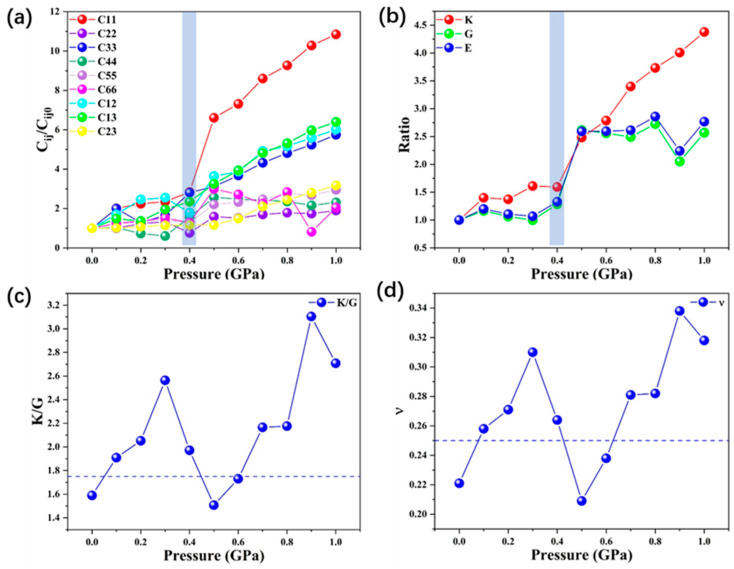
(**a**) Pressure dependences of elastic constants (*C_ij_*); (**b**) pressure dependences of mechanical moduli (*K, G, E*) for ZIF-4; (**c**) *K/G* ratio under various pressures (the horizontal dashed line denotes the value of *K/G* = 1.75); (**d**) Poisson’s ratio (*ν*) under various pressures (the horizontal dashed line denotes the value *ν* = 0.25).

Among the nine independent elastic constants, *C*_11_, *C*_22_, and *C*_33_ characterize the incompressibility along the x, y, and z directions, respectively. For the independent elastic constants obtained with GGA-PBE potential, the ratio of values calculated under high pressure to those under zero pressure, *C_ij_/C_ij_*(0 GPa), are depicted in Figure 3a. It can be seen from Table S2 and Figure 3a that before 0.4 GPa, C_22_ is the largest among *C*_11_*, C*_22,_ and *C*_33_. However, *C*_11_ becomes larger than the other two constants when the pressure increases above 0.4 GPa. This indicates that below 0.4 GPa, ZIF-4 is more incompressible along the b-axis (y direction). The pressure leads to changes in the structure so that it is more difficult to be compressed along the a-axis (y direction) above 0.4 GPa. *C*_11_*, C*_12,_ and *C*_13_ are more sensitive to the change in pressure than the other elastic constants. Additionally, the gradient of the constants increases between 0.4 GPa and 0.5 GPa, especially for *C*_11_, which indicates a fundamental change in the structure during this pressure range.

Similarly, the *K/K_0_*, *G/G_0_*_,_ and *E/E_0_* ratios are also calculated to ascertain the influence of pressure on elastic and mechanical properties, as illustrated in Figure 3b. The detailed values of *K, G*, and *E* are listed in Table S3. For the mechanical moduli of ZIF-4, the strengthening effect can be queued as *E > K > G* with increasing pressure. At different hydrostatic pressures, the shear modulus G is always smaller than bulk modulus *K*, which indicates the shear instability of orthorhombic ZIF-4. This is consistent with previous works that have claimed that the shear mode collapse is the main reason for pressure-induced amorphization [23,45,46,47].

The hardness and brittleness of the compounds are represented as the ratio (*K/G*) between bulk modulus and shear modulus, according to Pugh’s criterion [48]. Materials with good ductility usually have a high *K/G* value. The compound with a larger *K/G* ratio (>1.75) is usually ductile; otherwise, a *K/G* ratio < 1.75 is considered brittle. Cauchy pressure is another criterion used to determine structural brittleness/ductility. According to Pettifor’s rule [49], materials with large positive Cauchy pressures have more metallic bonds and thus become more ductile. If Cauchy pressures of the materials are strongly negative, they possess more angular bonds and thus exhibit more brittleness. The results of the *K/G* ratio and the Cauchy pressure of ZIF-4 are listed in Table S3 and shown in Figure 3c. It is shown that ZIF-4 is brittle at zero pressure, but it becomes ductile with external pressure going up from 0.1 to 0.4 GPa. It is followed by a decrease in *K/G* ratio that occurs in the pressure range of 0.3–0.5 GPa. The *K/G* ratio drops below 1.75 again at 0.5 GPa, indicating that ZIF-4 exhibits brittleness at this pressure. As the external pressure increases from 0.5 to 1 GPa, the *K/G* ratio again increases with external pressure. At pressures greater than 0.6 GPa, ZIF-4 undergoes a second brittle–ductile transition and the *K/G* ratio increases above the critical value of 1.75. 

Consistent with the trend of the change in the *K/G* ratio, the Cauchy pressure for ZIF-4 increases with pressure in both the 0–0.3 GPa and 0.4–1 GPa hydrostatic pressure change ranges. The trends of Cauchy pressure suggest an increase in the metallicity of bonding in the structure and ductility. However, the corresponding Cauchy pressure at 0.3 GPa is higher than at 0.4–0.8 GPa, suggesting that the change in bonding in the ZIF-4 structure with pressure is discontinuous. In conjunction with the structural changes discussed previously, the drastic volume change in the structure that occurs at 0.3–0.4 GPa essentially corresponds to a partial chemical bonding transition. 

In addition to the *K/G* ratio and Cauchy pressure, the Poisson’s ratio is also commonly used to evaluate the materials’ mechanical response under external stress. Typically, a larger Poisson’s ratio usually corresponds to good ductility. The value of *ν* for covalent materials is small (*ν* = 0.1), and for ionic materials, a typical value is 0.25. For central force solids, the value of *ν* is between 0.25 and 0.5 [50]. The calculated results of Poisson’s ratio for ZIF-4 are listed in Table S3 and shown in Figure 3d. These show a similar tendency as the ratio of *K/G*. The Poisson’s ratio increases from 0.221 in zero pressure to 0.310, until external pressure is reached at 0.3 GPa. Later, the Poisson’s ratio begins to decrease from 0.310 to 0.209 in the range of 0.3–0.5 GPa. Above 0.5 GPa, the Poisson’s ratio increases again from 0.209 to 0.318 with the pressure going up to 1 GPa. These results indicate that ionic features exist in the interatomic interactions and the structure is a central force solid.

**Figure 4:**
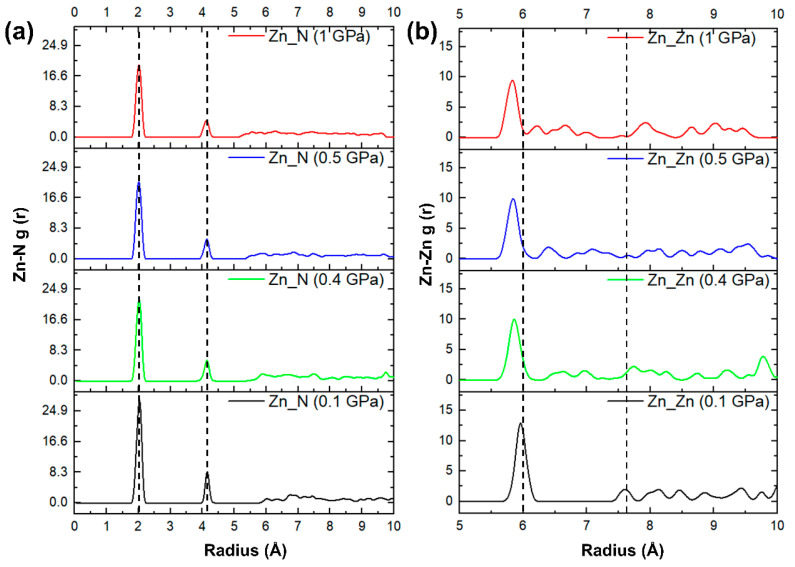
Pressure dependences of (**a**) Zn-N pairs and (**b**) Zn-Zn radius distribution function of ZIF-4.

The lattice parameters and mechanical properties discussed above indicate that for ZIF-4, 0.4 GPa is a “watershed” in hydrostatic pressure rises from 0 to 1 GPa. To reveal changes in the lattice parameters and mechanical properties near 0.4–0.5 GPa, the structures of ZIF-4 at 0.1, 0.4, 0.5, and 1 GPa were further characterized. The radial distribution function (RDF) in Figure 4 shows a mid-range structural shift at 0.4–0.5 GPa. When the pressure is smaller than 0.4 GPa, the compression of the structure corresponds to the contraction of the pores, and the difference in the distribution of the Zn-Zn bond lengths is slight. When the pressure increases to 0.4–0.5 GPa, the change in the form of the mid-range connections is reflected in the further reduction in Zn-Zn bonds. The Zn-Zn bond lengths at 0.9 GPa and above do not change further than at 0.5 GPa. In the short-range structure presented by the Zn-N pair RDF in Figure 4a, there is little change under different pressures. From this, we deduce that the anomaly of decreasing ductility and plasticity of the ZIF-4 structure with increasing pressure is due to the transformation in the way the [ZnN_4_] tetrahedra are connected. The change in the mid-range order explains the sudden change in the mechanical properties of silicates with similar [SiO4] tetrahedral network structures [33,51].

## 3. Discussion

In this work, we have investigated the structural evolution and mechanical properties of ZIF-4 at low hydrostatic pressures (<1 GPa). For this purpose, the atomic structure and elastic constants of ZIF-4 have been investigated concerning external pressure ranging from 0 GPa to 1 GPa using the DFT calculation method. The phase stability, mechanical moduli, and Debye temperature are discussed. The elastic constants, mechanical moduli, and Debye temperature showed increases with increasing pressure. Furthermore, we analyzed the geometrical characteristics of the structure and found that the form of the connection of the [ZnN_4_] tetrahedra in the structure changes at 0.4 GPa. The pressure leads to a twisting of the Im ligands. This mid-range structural shift leads to the creation of intra-molecular forces between ligands, in addition to a reduction in free volume. Affected by the new mid-range structures, anomalies in the ZIF-4 elastic constants are produced at around 0.4 GPa. These include sudden increases in elastic moduli, negative linear compressibility, and the transition from ductile to brittle as reflected in *K/G*. This change in mid-range structure due to the twisting of polyhedral units has also been investigated in other simulations of the mechanical properties of tetrahedral network structures. An example is the effect of the [SiO_4_] tetrahedral connection on the anomalous mechanical properties of SiO_2_ glasses at extreme high pressures [33]. This suggests that it is instructive to study changes in the mid-range local structure of amorphous structures.

It is worth noting that errors between theoretical and experimental values are inevitable due to limitations in the accuracy of the theoretical simulation method and the size of the model used. Our analysis is based on the central idea that varied mechanical properties correlate with mid-range structural evolution. For amorphous or other structures that are not at the lowest point on the energy landscape map, the greatest difference in static structure from their crystalline state is reflected in the mid-range order. This difference causes the response of the structure under stress or other external fields to diverge from that of the crystalline state. For the crystal–amorphous transition in MOF and other complex systems, larger-scale ab initio simulations based on more advanced computers or algorithms are the focus of further research. Another possible research prospect is the establishment of correlations across multi-scale simulations, as has been completed in catalytic simulations [52,53]. By using structural features of smaller spatial and temporal scales as input, the establishment of correlations between simulation tools at different scales will have a positive effect on the experimental–simulation combination. Further exploration of the effects of different polyhedral unit connections on structural properties at different length scales will help to establish a clearer link between atomic-scale simulations and macroscopic-scale performance.

## 4. Materials and Methods

The first-principles method, based on density functional theory (DFT), was used in the geometry optimization and total energy calculation of the structures of ZIF-4. The original ZIF-4 structure was obtained from The Cambridge Crystallographic Data Centre (CCDC) [54] with the number VEJYUF01 [55], and the solvent molecules were removed. The projector augmented wave (PAW) method was implemented in the Vienna ab initio Simulation Package (VASP) code [56]. The electronic exchange and correlation potential were expressed using the Perdewe–Burkee–Ernzerh (PBE) version of the generalized gradient approximation (GGA) [57]. The cut-off energy for the plane wave basis set was 600 eV for all structures. Structural relaxations were performed to a tolerance of 1 × 10^−7^ eV/atom in the total energy, yielding average forces of 1 × 10^−3^ eV/Å. The stress level of the final equilibrium structure was less than 0.1 GPa. The relaxation of all the structures imposed no restrictions on the volume and lattice vectors. Since the unit cells were large, containing nearly 400 hundred atoms, only one K-point at Γ (0, 0, 0) was used.

The effects of external pressure were enforced by the stress tensor (PSTRESS keyword) corresponding to a selected pressure value in the range between 0 GPa and 1 GPa. Energies and enthalpies were calculated at multiple pressure points starting from the experimental structure, but both the atomic positions and the unit cell parameters relaxed during energy optimization under the corresponding pressures.

The single-crystal elastic constants, *C_ij_*, of the elasticity matrix (tensor) were then computed by performing six finite distortions of the lattice and deriving the elastic constants from the strain–stress relationship with the code in VASP [58,59]. 

As is well known, the ZIF-4 phase belongs to the orthorhombic crystal which has nine independent elastic constants, i.e., *C*_11_, *C*_22_, *C*_33_, *C*_44_, *C*_55_, *C*_66_, *C*_12_, *C*_13_, and *C*_23_. From the results of the calculated *Cij*, the isotropic bulk modulus (*K*), shear modulus (*G*), Young’s modulus (*E*), and Poisson’s ratio (*υ*) of the ZIF-4 phase were determined with the Voigte–Reusse–Hill (VRH) averaging scheme [60]. For the orthorhombic system, the Vogit and Reuss bounds of *K* and *G* can be expressed as follows [61,62]:

(4) $$K_{V}=\frac{1}{9}\left[C_{11}+C_{22}+C_{33}+2\left(C_{12}+C_{13}+C_{23}\right)\right]\;$$

(5) $$K_{R}={\left(S_{11}+S_{22}+S_{33}+2\left(S_{12}+S_{13}+S_{23}\right)\right)}^{-1}\;$$

(6) $$G_{V}=\frac{1}{15}\left(C_{11}+C_{22}+C_{33}-C_{12}-C_{13}-C_{23}\right)+\frac{1}{5}\left(C_{44}+C_{55}+C_{66}\right)\;$$

(7) $$G_{R}=15\left[4(S_{11}+S_{22}+S_{33}\right)+3\left(S_{44}+S_{55}+S_{66}\right)-4\left(S_{12}+S_{13}+S_{23}\right){]}^{-1}\;$$

where *S_ij_* represents the elastic compliance matrices. The Hill bulk modulus (*K_H_*) and shear modulus (*G_H_*) can be given as:

(8) $$K_{H}=\frac{1}{2}\left(K_{R}+K_{V}\right)\;$$

(9) $$G_{H}=\frac{1}{2}\left(G_{R}+G_{V}\right)\;$$

The Young’s modulus (*E*) and Poisson’s ratio (*ν*) of the polycrystalline aggregate can be calculated via *K_H_* and *G_H_*, as follows:

(10) $$E_{VRH}=\frac{9K_{H}G_{H}}{3K_{H}+G_{H}}\;$$

(11) $$v_{VRH}=\frac{3K_{H}-2G_{H}}{2\left(3K_{H}+G_{H}\right)}\;$$

In order to comprehensively estimate the elastic anisotropy of these materials, the universal elastic anisotropy index *A^U^* is adopted, accounting for both the shear and bulk contributions [63,64]:

(12) $$A^{U}=5\frac{G^{V}}{G^{R}}+\frac{K^{V}}{K^{R}}-6\;$$

The Debye temperature (*θ_D_*) of materials plays an important role in many physical properties of solids, such as specific heat, elastic stiffness constants, and melting temperature. The experimental value of a solid can usually be calculated from the sound velocity. The Debye temperature of materials can be deduced from elastic constants. The Debye temperature of ZIF-4 was calculated using the average sound velocity (*V_m_*) with the following equation [65]:

(13) $$\theta_{D}=\frac{h}{k}{\left[\frac{3n}{4\pi }\left(\frac{N_{A}\rho }{M}\right)\right]}^{1/3}V_{m}\;$$

where *h* is Planck’s constant, *k* is Boltzmann’s constant, *N_A_* is Avogadro’s number, *ρ* (=*M/V*) is the density, *M* is the molecular weight, and *n* is the number of atoms per formula unit. The average wave velocity (*V_m_*) in the polycrystalline material can be calculated with the following equation:

(14) $$V_{m}={\left[\frac{1}{3}\left(\frac{2}{V_{t}^{3}}+\frac{1}{V_{I}^{3}}\right)\right]}^{-1/3}\;$$

where *V_t_* and *V_l_* are the transverse and longitudinal sound velocity, respectively, which are obtained from the values of Hill’s bulk modulus *K* and shear modulus *G* from Navier’s equation [65]:

(15) $$V_{t}=\sqrt{\frac{G}{\rho }}c\;$$

(16) $$V_{I}=\sqrt{\left(K+\frac{4}{3}G\right)/\rho }\;$$

## Figures and Tables

**Table 1:** Lattice parameter volume per unit cell of ZIF-4 under various pressures.

Pressure (GPa)	*a* (Ǻ)	*b* (Ǻ)	*c* (Ǻ)	*V* (Ǻ^3^)
0	15.350	15.811	18.519	4494.540
Ref [15]	15.403	15.459	18.408	4383.220
Ref [39]	15.370	15.080	18.610	4313.418
0.1	15.570	15.789	18.161	4464.605
0.2	15.546	15.539	17.970	4341.000
0.3	15.327	15.312	18.000	4224.366
0.4	15.958	15.544	14.631	3629.236
0.5	16.198	14.166	15.322	3515.799
0.6	16.160	14.042	15.253	3461.191
0.7	16.165	13.903	15.153	3405.515
0.8	16.194	13.805	15.025	3358.962
0.9	16.198	13.661	14.993	3317.664
1	16.215	13.582	14.888	3278.816

## Data Availability

Input files of simulation are available from the authors.

## Supplementary Materials

The following supporting information can be downloaded at: https://www.mdpi.com/article/10.3390/molecules28010022/s1. Table S1. The pressure dependence of density *ρ*, average sound velocity (*V_t_*), longitudinal sound velocity (*V_I_*), average wave velocity (*V_m_*), and Debye temperature (*θ_D_*) of ZIF-4. Table S2. The calculated elastic constants *C_ij_* (GPa). Table S3. The calculated Cauchy pressure (*C_12_-C_44_*), elastic modulus (GPa), Poisson’s ratio *υ*, and anisotropy factor A of ZIF-4 under pressure from 0 GPa to ~1 GPa.

## Sample Availability

Samples of the compounds are available from the authors.
