# Supplementary material for: The Atomic Structure and Mechanical Properties of ZIF-4 under High Pressure: Ab Initio Calculations
Source: Molecules. 2022 Dec 20;28(1):22. doi: 10.3390/molecules28010022 (PMC9821817; doi:10.3390/molecules28010022)
Supplement: Supplementary file 1 [file molecules-28-00022-s001.zip › molecules-2031610-supplementary.pdf]

Table S1. The pressure dependence of density ϱ, average sound velocity (Vt), longitudinal sound velocity (VI), average wave velocity (Vm), and Debye temperature (θD) of ZIF-4

Pressure(GPa)	$\rho\times10^3$ (kg/m^3)	V_t (m/s)	V_l (m/s)	V_m (m/s)	Θ_D (K)
0	1.179	970.252	1658.603	1075.901	125.743
0.1	1.187	1043.957	1879.862	1162.748	136.198
0.2	1.221	983.338	1809.171	1097.035	129.711
0.3	1.255	939.934	1855.551	1053.608	125.712
0.4	1.461	988.299	1796.523	1101.566	138.257
0.5	1.508	1387.951	2339.191	1537.055	194.968
0.6	1.532	1362.046	2383.967	1513.500	192.982
0.7	1.557	1333.538	2494.604	1489.523	190.952
0.8	1.578	1384.586	2593.879	1546.702	199.200
0.9	1.598	1193.934	2515.154	1343.182	173.703
1	1.617	1328.174	2669.956	1490.409	193.501

Table S2.The calculated elastic constants C_{ij} (GPa)

Pressure(GPa)	C_{11}	C_{22}	C_{33}	C_{44}	C_{55}	C_{66}	C_{12}	C_{13}	C_{23}
0	1.723	3.721	1.770	0.747	1.746	2.413	0.984	1.522	1.770
0.1	3.466	3.738	3.541	0.777	2.128	3.015	1.685	2.264	1.807
0.2	3.869	4.602	2.281	0.549	2.324	3.305	2.420	2.076	1.906
0.3	4.050	4.940	2.919	0.455	2.435	3.668	2.510	2.983	2.039
0.4	4.897	2.854	4.970	1.169	2.288	3.095	1.765	3.580	2.056
0.5	11.383	5.928	5.517	1.919	3.860	7.229	3.590	4.956	2.066
0.6	12.600	5.652	6.516	1.855	4.073	6.563	3.809	5.993	2.649
0.7	14.822	6.335	7.659	1.821	4.309	5.438	4.838	7.334	3.727
0.8	15.973	6.659	8.522	1.764	4.834	6.850	5.083	8.095	4.335
0.9	17.710	6.491	9.272	1.615	4.686	1.975	5.508	9.089	4.962
1	18.683	7.076	10.172	1.728	5.152	4.956	5.919	9.739	5.625

Table S3. The calculated Cauchy pressure (C12-C44), elastic modulus (GPa), Poisson's ratio υ, and anisotropy factor A of ZIF-4 under pressure from 0GPa~1GPa

Pressure(GPa)	C_{12}-C_{44}	*K*	*G*	*E*	*K/G*	*υ*	A
0	0.237	1.764	1.110	2.753	1.589	0.221	2.357
0.1	0.908	2.471	1.294	3.305	1.909	0.258	2.078
0.2	1.871	2.423	1.181	3.047	2.052	0.271	4.301
0.3	2.055	2.842	1.109	2.943	2.564	0.310	8.176
0.4	0.597	2.812	1.427	3.661	1.971	0.264	2.197
0.5	1.671	4.378	2.905	7.136	1.507	0.209	2.405
0.6	1.954	4.916	2.841	7.147	1.730	0.238	2.303
0.7	3.017	5.996	2.768	7.196	2.166	0.281	2.004
0.8	3.319	6.584	3.026	7.871	2.176	0.282	2.603
0.9	3.893	7.071	2.278	6.171	3.104	0.338	1.874
1	4.191	7.723	2.852	7.618	2.708	0.318	2.379
